# Embedding the Use of Patient Multimedia Educational Resources Into Cardiac Acute Care: Prospective Observational Study

**DOI:** 10.2196/54317

**Published:** 2024-07-18

**Authors:** Anastasia Hutchinson, Damien Khaw, Annika Malmstrom-Zinkel, Natalie Winter, Chantelle Dowling, Mari Botti, Joanne McDonall

**Affiliations:** 1 Centre for Quality and Patient Safety Research—Epworth Partnership Institute of Health Transformation, Faculty of Health Deakin University Melbourne Australia; 2 School of Nursing and Midwifery Faculty of Health Deakin University Geelong Australia

**Keywords:** patient participation, digital technology, mHealth, mobile health, app, apps, digital health, smartphone, smartphones, multimedia, patient education, education, educational, educate, patient engagement, nursing, cardiac surgery, cardiology, cardiac, cardio, CCU, cardiac care unit, CCC, complex cardiac care, coronary care nursing, nurse, nurse, COVID-19, SARS-COV-2, Coronavirus, severe acute respiratory syndrome, Coronavirus infections, novel Coronavirus

## Abstract

**Background:**

Multimedia interventions may play an important role in improving patient care and reducing the time constraints of patient-clinician encounters. The “MyStay Cardiac” multimedia resource is an innovative program designed to be accessed by adult patients undergoing cardiac surgery.

**Objective:**

The purpose of this study was to evaluate the uptake of the MyStay Cardiac both during and following the COVID-19 pandemic.

**Methods:**

A prospective observational study design was used that involved the evaluation of program usage data available from the digital interface of the multimedia program. Data on usage patterns were analyzed for a 30-month period between August 2020 and January 2023. Usage patterns were compared during and following the lifting of COVID-19 pandemic restrictions. Uptake of the MyStay Cardiac was measured via the type and extent of user activity data captured by the web-based information system.

**Results:**

Intensive care unit recovery information was the most accessed information, being viewed in approximately 7 of 10 usage sessions. Ward recovery (n=124/343, 36.2%), goal (n=114/343, 33.2%), and exercise (n=102/343, 29.7%) information were routinely accessed. Most sessions involved users exclusively viewing text-based information (n=210/343, 61.2%). However, in over one-third of sessions (n=132/342, 38.5%), users accessed video information. Most usage sessions occurred during the COVID-19 restriction phase of the study (August 2020-December 2021). Sessions in which video (*P*=.02, *phi*=0.124) and audio (*P*=.006, *phi*=0.161) media were accessed were significantly more likely to occur in the restriction phase compared to the postrestriction phase.

**Conclusions:**

This study found that the use of digital multimedia resources to support patient education was well received and integrated into their practice by cardiac nurses working in acute care during the COVID-19 pandemic. There was a pattern for greater usage of the MyStay Cardiac during the COVID-19 pandemic when access to the health service for nonfrontline, essential workers was limited.

## Introduction

In 2020, a total of 14,408 cardiothoracic operations were performed in 39 specialist cardiac surgery centers in Australia [1]. Open-heart surgery that involves sternotomy includes an intensive care unit (ICU) stay for 24-72 hours, followed by 5-7 days in a specialist cardiac ward for treatment optimization and recovery. Optimal recovery outcomes following major surgery require patients to be informed about and engaged in their care [2]. When patients are engaged in their own care, there is evidence to suggest that their recovery is both informed and safer [3,4]. The therapeutic relationship that develops between patients and nurses in acute care is the foundation for patient-centered care, which is required by the National Safety and Quality Health Service standards [5].

The journey of patients who underwent cardiac surgery through the hospital system begins prior to and on admission and is facilitated by a multidisciplinary team, including cardiac nurses, who educate patients about what can be expected during their ICU and cardiac ward admission. To aid and support patient recovery post cardiac surgery, administration of adequate analgesics to expedite participation in chest physiotherapy and early mobility [6-10] is a key recovery intervention facilitated by the multidisciplinary team [2,11]. Despite its importance, patient education can be time-consuming in acute care when the acuity of illness, an unfamiliar environment, and the effects of medications and treatment impact patients’ information recall [12].

Multimedia interventions may play an important role in improving patient comprehension and reducing the time constraints of patient-clinician encounters [13,14]. The use of multimedia interventions in patient care may help minimize time requirements that are usually needed for clinicians to provide patient education [12]. Several studies have shown that patients learn and retain information better when they are provided with both visual and verbal information presented together [15,16]. A major strength of using multimedia resources is the consistency of messaging and the fact that patients can access the resources at their convenience, which aids in the recall and retention of key information [14,17]. This is particularly important in the early stages of acute recovery when patients are busy with procedures and treatments that affect cognition. Having a resource for patients that is available when they have the opportunity, is easy to use, can be delivered when the patient is ready, and may help mitigate inequalities related to reading ability, viz, text-only patient education, are also important considerations [12,17].

It is important to evaluate the feasibility of nurses using digital multimedia to promote patient engagement and participation in care. Furthermore, with the increasing use of digital technology to promote patient participation in care [14,18,19], it is important to review the implementation of such technology in the postoperative setting [20]. The “MyStay Cardiac” multimedia program is an innovative program designed to be accessed by adult patients undergoing cardiac surgery. The program can be accessed by patients independently or with the assistance of nurses in the ICU, coronary care unit and cardiac ward to monitor their pain intensity score and mobility improvement while in hospital, recovering from cardiac surgery. The aim of the MyStay Cardiac program is to support the recovery of patients being admitted for cardiac surgery in the pre- and postoperative phases [17].

The MyStay Cardiac multimedia program was implemented at a private metropolitan hospital in Melbourne during the COVID-19 pandemic in July 2020. During 2020, with the escalating COVID-19 pandemic, the provision of nonmandatory staff education in hospitals was limited [21], and as such, a passive diffusion approach to implementation was used [22]. Evidence suggests that both patients and staff require structured education and support to implement such a program successfully [23]. It is therefore both important and interesting to gain an understanding of how successful the program has become without ongoing structured facilitation in the clinical practice setting [22].

The purpose of this study was to evaluate the uptake of a multimedia intervention, MyStay Cardiac, both during and following the COVID-19 pandemic. The specific aims of this study are provided in Textbox 1.

**Specific aims of this study.**

- To explore the uptake and use of different modules and functionalities within the Cardiac MyStay multimedia program.
- To compare use of the MyStay Cardiac during periods of restricted access to hospitals due to the COVID-19 pandemic (August 2020-December 2021) to use once the COVID-19 restrictions were no longer in place (January 2022-January 2023).

## Methods

### Study Design

A prospective observational study design was used that involved the evaluation of program utilization data available from the digital interface for the multimedia program. Sampling was consecutive; all patients on the ward were offered the opportunity to use the MyStay app during their stay and to participate in this research. The inclusion criterion was as follows: patients on the cardiac ward who used the MyStay app. Patients who did not use the MyStay app were excluded from the study. Though not formally excluded on the basis of such characteristics, it is possible that adverse health events, language, or cognition issues may have precluded some patients from using the MyStay app and thus from participating in the study.

### Study Site

The study was conducted at a large private health service in Victoria, Australia.

### MyStay Cardiac Multimedia Resources

The MyStay Cardiac multimedia program combines text, audio, video, and 3D animations to deliver information to patients and their families to enhance their understanding of, and participation in, postoperative care, meeting recovery goals, and discharge planning [17]. The web-based platform allows patients to access MyStay both prior to and following their surgery. Content within the cardiac surgery app is organized into the following modules: (1) ICU stay, (2) ward stay, (3) exercise and rehabilitation, (4) pain management, (5) keeping you safe (preventing complications), and (6) preparing for discharge. The program can be accessed by patients independently or with the assistance of nurses in the ICU, coronary care unit, and cardiac ward to monitor their pain intensity score and mobility improvement whilst in hospital, recovering from cardiac surgery. During the study period, MyStay pain intensity scores were used for informational purposes only, that is, for patients’ records and understanding, to encourage participation in their care, and not as a part of formal, clinical assessments of pain.

### Implementation of the MyStay Cardiac at the Study Site

The MyStay Cardiac program was made available to cardiac nurses and allied health staff in July 2020, following a web-based education session. During this session, each module of the MyStay Cardiac was demonstrated to the cardiac nurse educators, cardiac ward nurses, and physiotherapy team. Hospital information technology administrators made the program accessible to the wide-screen, bedside computer terminals located in all patients’ rooms. The clinicians were provided with a generic login and password so they could access the MyStay resources and familiarize themselves with the content. Clinicians were then encouraged to integrate the MyStay Cardiac resources into their interactions with patients undergoing cardiac surgery at the study site. Following this initial education session, further structured implementation by the research team was not possible in 2020-2021 due to state government restrictions on nonessential staff attending hospital sites during the COVID-19 pandemic in Melbourne, Australia [21]. There was no change to the physical infrastructure and availability of the app to patients either before or following COVID-19 restrictions. Further, in addition to using the bedside computers, patients could also access the app via their personal devices (eg, smartphones, tablets, and laptops).

To verify how the MyStay Cardiac resources were being used, 3 cardiac nurses (2 cardiac educators and 1 nurse manager) were interviewed in late 2021 and indicated high levels of satisfaction with the MyStay Cardiac resources, stating they were accessible, relevant to care delivery both before and after the cardiac survey, and a useful adjunction to the usual cardiac education they provided to patients during their admission. The cardiac nurse educators reported predominantly using the MyStay Cardiac resources in structured preoperative patient education sessions provided on the day prior to surgery. The availability of animations demonstrating what to expect during an ICU admission provides an alternative to an in-person ICU visit the day before surgery. As part of the standard routine of the cardiac wards, structured postoperative education sessions focusing on preparation for discharge, care, and recovery at home are provided regularly on Wednesdays.

### Uptake and Usage of the MyStay Cardiac

Uptake of the MyStay Cardiac was measured via the type and extent of user activity data captured by the web-based information system: page visits; button clicks; audio starts and stops; and video starts and stops. Records were outputted into a spreadsheet. Specific data fields included: activity day of the week and date; activity duration; and activity location and type (Multimedia Appendix 1).

To aid the analysis of activity data, MyStay Cardiac usage records were partitioned into discrete usage sessions. A MyStay session was defined as an unbroken sequence of user activity, with gaps between activities not exceeding 15 minutes. The duration of an activity was calculated by the length of time between 2 consecutive activities. As no usage followed the final activity of each MyStay session, we substituted this value with the total mean activity duration for the corresponding MyStay session. Each session was categorized according to the combination of MyStay material accessed by users. Categories were as follows: (1) ICU stay information only (ICU and day 1 or 2); (2) ward stay information only (ward and day 3+); (3) exercise information only; (4) ICU and exercise information; (5) ward and exercise information; (6) ICU and ward information; and (7) all the above.

To evaluate the impact of the COVID-19 lockdowns that restricted access to health services by nonessential personnel, the following time periods were defined: restricted access to acute hospitals, August 2020-December 2021, and unrestricted access, January 2022-January 2023.

### Data Analysis

Data summaries were performed using descriptive statistics. The normality of continuous variables was assessed via visual inspection of histograms: total items clicked; average activity duration; and total session length. Where continuous variables were non-normal, central tendency and spread were reported according to the median and IQR. Otherwise, means and SDs of continuous data were reported.

Strengths of association between categorical variables measuring app usage were evaluated using chi-square tests of Independence: ICU recovery material accessed; ward recovery material accessed; goals accessed; exercise material accessed; pain record accessed; video accessed; audio accessed; and access during patient education days (Wednesdays). Where contingency tables were 2×2, a continuity correction was applied to chi-square analyses. Between-group comparisons of continuous data were performed using Kruskal-Wallis H tests, with post hoc analyses performed using Mann-Whitney *U* tests. Data were summarized and analyzed in SPSS Statistics (version 29; IBM Corp).

#### Ethical Considerations

This paper reports on a specific component within a larger observational program of research on the MyStay multimedia app. Ethics and research governance approval for the larger study was given by the Human Research Ethics Committee of the participating university (#2020-053) and the Research Governance Unit of the hospital site, respectively. Participants engaged in the formal data collection methods within the larger study are required to give informed written consent to participate. However, the data collected as part of the protocols reported in this study were only anonymous application usage data, routinely collected by the technology platform as part of usual care. Consent to participate in these particular protocols was implied; the MyStay app indicates in its terms of service that users agree that de-identified, aggregated usage data may be used for the purposes of conducting health-related research.

## Results

### Principal Findings

Data on usage patterns of the MyStay Cardiac resources was analyzed for a 30-month period between August 2020 and January 2023.

Usage outcomes of MyStay Cardiac are reported for the 2 study phases in Table 1. ICU recovery information was the most accessed information, being viewed in approximately 7 in 10 usage sessions. Ward recovery (n=124/343, 36.2%), goal (n=114/343, 33.2%), and exercise (n=102/343, 29.7%) information were routinely accessed. However, access to the patient pain record was very infrequent (n=20/343, 5.8% of sessions). Most sessions involved users exclusively viewing text-based information (n=210/343, 61.2%). However, in over a third of sessions (n=132/342, 38.5%), users accessed video information. Further, over a third of sessions occurred were on patient education that was provided to patients on Wednesday during their acute care admission. The focus of these sessions was on recovery following surgery and rehabilitation following acute care discharge. This finding is in line with the report from the cardiac nurse educators that they predominantly used the MyStay Cardiac for pre- and postoperative education sessions.

**Table 1:** Usage of the MyStay Cardiac during COVID-19 restriction and post–COVID-19 restriction phases (August 2020-January 2023).

			COVID-19 restrictions			Total		*P* values
Use of MyStay Cardiac			Restriction phase^a^		Post-restriction phase^b^			
Number of usage sessions, n (%)			213 (62.1)		130 (37.9)	343 (100)		—^c^
**Type of material accessed**								
	ICU^d^ recovery, n (%)	141 (66.2)		96 (73.8)		237 (69.1)	.17^e,f^	
	Ward recovery, n (%)	81 (38)		43 (33.1)		124 (36.2)	.42^e,f^	
	Goals, n (%)	77 (36.2)		37 (28.5)		114 (33.2)	.18^e,f^	
	Exercise, n (%)	65 (30.5)		37 (28.5)		102 (29.7)	.78^e,f^	
	Pain record, n (%)	17 (8)		3 (2.3)		20 (5.8)	.05^e,f^	
**Accessed audio-visual material**								
	Video, n (%)	92 (43.2)		40 (30.8)		132 (38.5)	.03^e,f^	
	Audio, n (%)	21 (9.9)		2 (1.5)		23 (6.7)	.006^e,f^	
**Access day**								
	Patient education days (Wednesday), n (%)	81 (38)		45 (34.6)		126 (36.7)	.60^e,f^	
	Total items clicked, median (IQR)	13 (0.0-27.5)		9.5 (0.0-24.5)		12 (0.0-26.0)	.45^g^	
	Activity duration (seconds); median (IQR)	13 (2.5-23.5)		18 (6.5-29.5)		15 (4.0-26.0)	.11^g^	
	Total session length (minutes); median (IQR)	181 (0-586.5)		210 (0.0-810.0)		192 (0.0-706.0)	.67^g^	

^a^Restriction phase August 2020-December 2021.

^b^Post-restriction phase January 2022-January 2023.

^c^Not applicable.

^d^ICU: intensive care unit.

^e^Chi-square test of independence.

^f^Continuity correction applied.

^g^Mann-Whitney *U* test.

### Impact of COVID-19 Pandemic Restrictions on MyStay Cardiac Usage

Most usage sessions occurred during the COVID-19 restriction phase of the study (August 2020-December 2021). Sessions in which video (*P*=.02, *phi*=0.124) and audio (*P*=.006, *phi*=0.161) media were accessed were significantly more likely to occur in the restriction phase compared to the post-restriction phase. Information on ICU recovery was the most used module, with MyStay Cardiac being accessed by users in 237/343 (69.1%) usage sessions. This pattern of usage reflects the use of the MyStay Cardiac for preoperative education on the day of admission to the hospital. No significant associations were present between the study phase and whether system access occurred on patient education days or whether ICU recovery, ward recovery, goal, exercise, or pain record material was accessed during sessions. Furthermore, no significant differences in total items clicked, activity duration, and total session length between the restriction and postrestriction phases were found.

### Usage Patterns

Associations and differences in usage outcomes between the 3 session types (rapid view, content exploration and in-depth look) are reported in Table 2. Chi-square tests of independence indicated statistically significant associations between patterns of MyStay use and the type of MyStay material accessed: ICU recovery (*P*<.001, Cramer V=0.373); ward recovery (*P*<.001, Cramer V=0.289); goals (*P*<.001, Cramer V=0.330); exercise (*P*<.001, Cramer V=0.531); audio content (*P*<.001, Cramer V=0.250); and pain records (*P*=.009, Cramer V=0.166). Examination of adjusted standardized residuals revealed that content exploration and in-depth look users were significantly more likely than rapid view users to access ICU recovery, ward recovery, and goal information (*z*_res_≥2.0). In-depth users were significantly more likely than rapid view and content exploration users to access exercise, audio, and pain recording content (*z*_res_≥2.0). No statistically significant association was found between patterns of MyStay use and use of the applications during the inpatient education sessions provided prior to hospital discharge.

**Table 2:** Usage of the MyStay Cardiac by session type: rapid views, content exploration, and in-depth looks.

		Session type		
		Rapid view	Content exploration	In-depth look
Number of usage sessions, n (%)		154 (44.9)	102 (29.7)	87 (25.4)
**Type of material accessed**				
	Home page only, n (%)	40 (26)	0 (0)	0 (0)
	ICU^a^ recovery, n (%)	77 (50)	87 (85.3)^b^	73 (83.9)^b^
	Ward recovery, n (%)	32 (20.8)	49 (48)^b^	43 (49.4)^b^
	Goals, n (%)	25 (16.2)	45 (44.1)^b^	44 (50.6)^b^
	Exercise, n (%)	9 (5.8)	36 (35.3)	57 (65.5)^b^
	Audio content, n (%)	1 (0.6)	8 (7.8)	14 (16.1)^b^
	Pain record, n (%)	3 (1.9)	7 (6.9)	10 (11.5)^b^
**Access day**				
	Patient education days (Wednesday), n (%)	53 (34.4)	38 (37.3)	35 (40.2)
	Total items clicked, median (IQR)	5 (1.9-8.1)	20 (6.1-33.9)^c^	44 (7.0-81.0)^c^
	Activity duration (seconds); median (IQR)	6.5 (0.5-12.5)	23.5 (11.5-35.5)^c^	22 (10.0-34.0)^c^
	Total session length (minutes); median (IQR)	33 (6.1-59.9)	524.5 (233.3-815.8)^c^	896 (399.0-1393.0)^c^

^a^ICU: intensive care unit.

^b^Standardised residuals of Chi-square test of Independence indicate statistically significant contribution of cell (*z*_res_≥2.0).

^c^Post-hoc Mann-Whitney *U* test, *P*<.001.

There was a significant effect of the patterns of MyStay use on the total number of items clicked (*H*=185.58, *P*<.001, *η*^2^=0.53), mean activity duration (*H*=78.072, *P*<.001, *η*^2^=0.22), and total session length (*H*=241.861, *P*<.001, *η*^2^=0.70). Post-hoc Mann-Whitney *U* tests revealed that content exploration and in-depth sessions involved significantly more total items clicked, and significantly higher mean activity and total session durations (all *P* values<.001).

**Figure 1:**
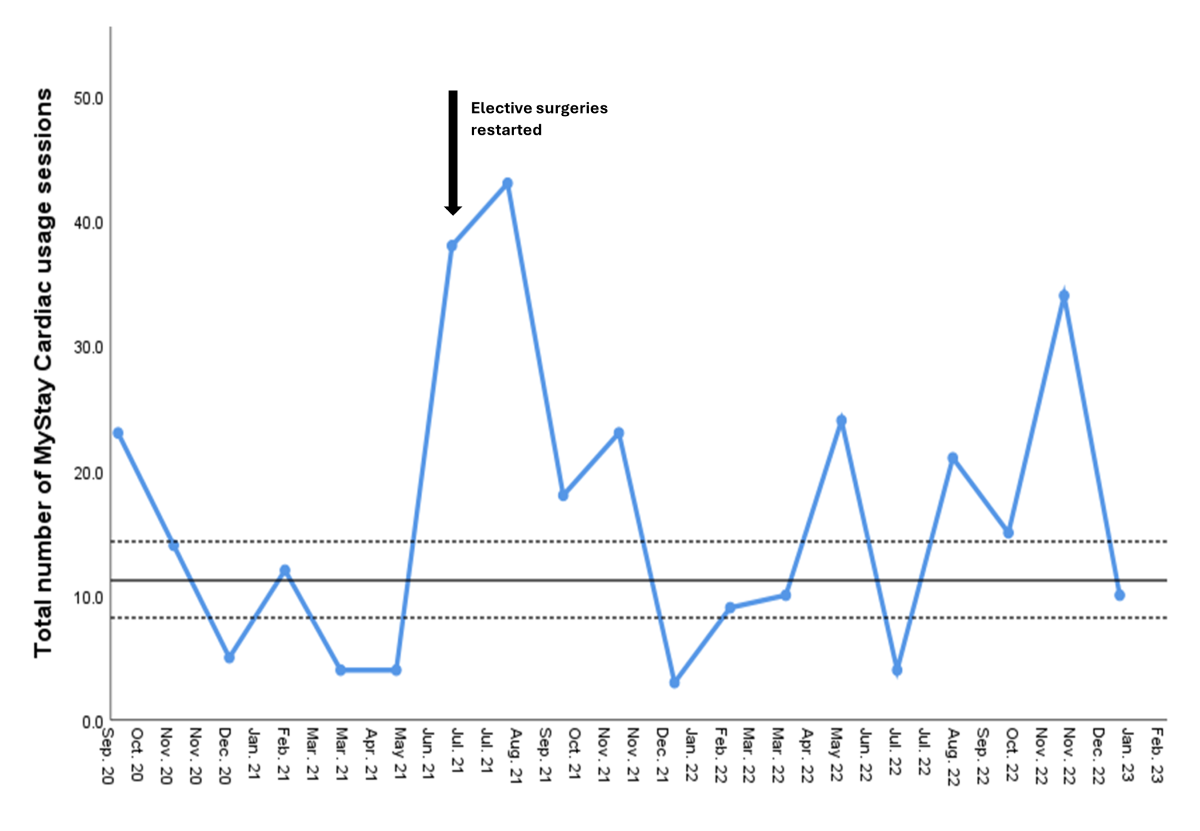
MyStay Cardiac—total number of usage sessions per month. Dotted lines denote the 95% CI around the mean.

**Figure 2:**
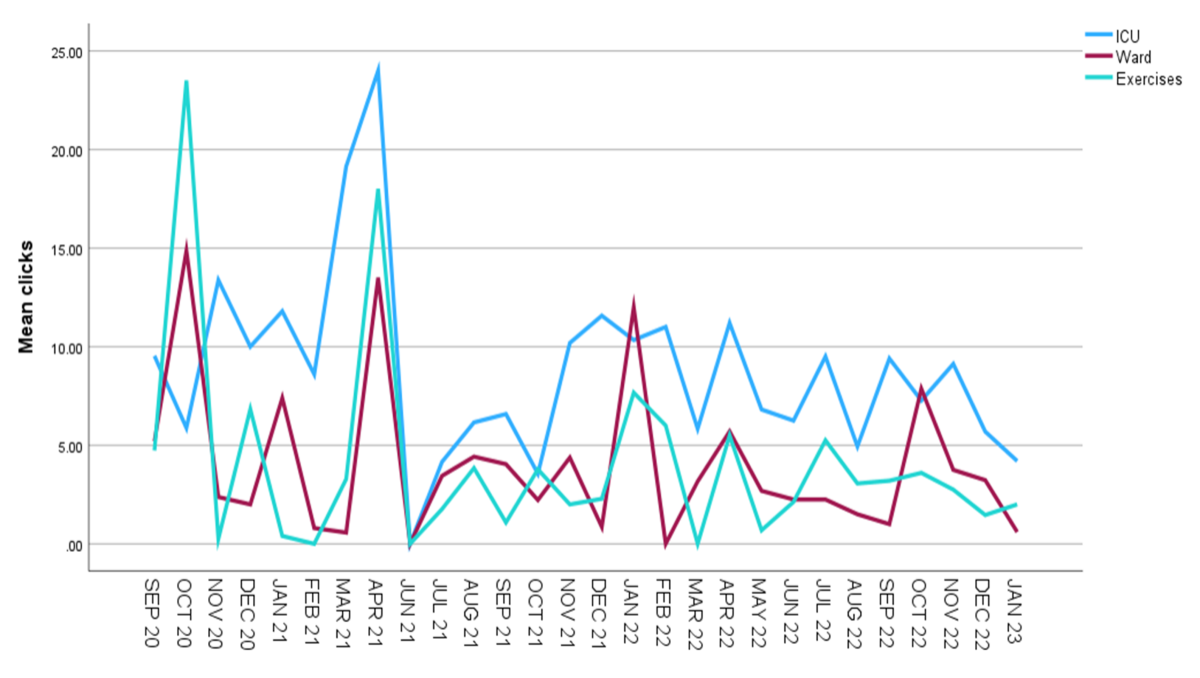
MyStay Cardiac—mean number of clicks within usage sessions per month. ICU: intensive care unit.

**Figure 3:**
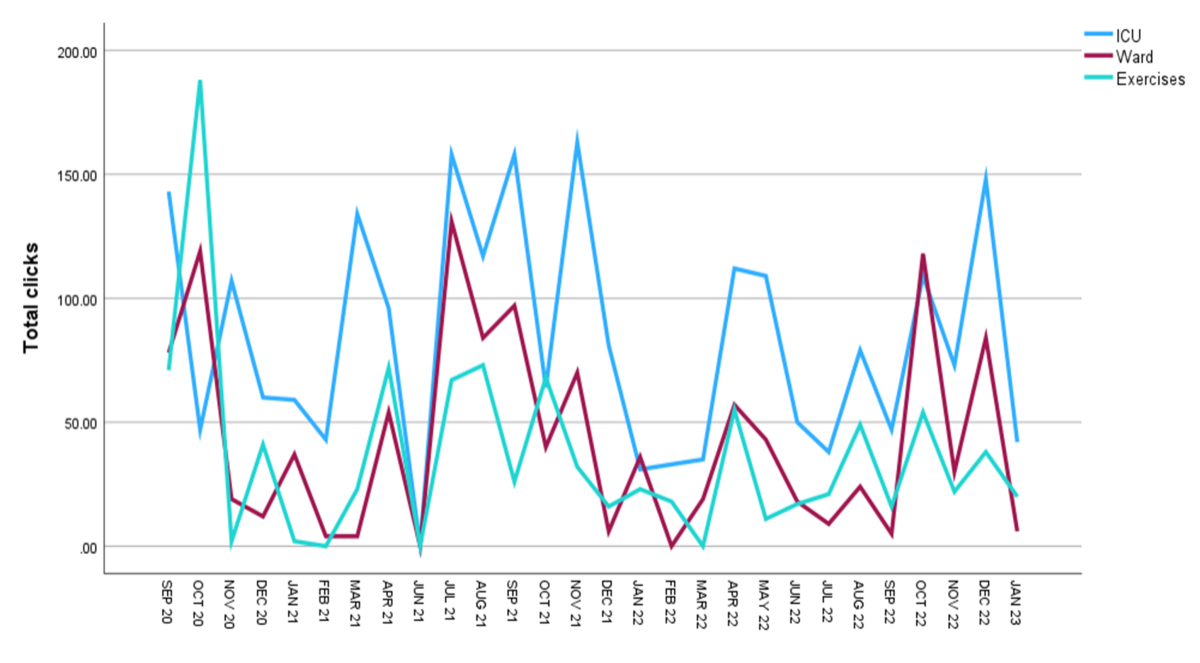
MyStay Cardiac—total website clicks per month. ICU: intensive care unit.

Figures 1-3 describe the total usage sessions per month, the mean number of website clicks per month, and total clicks per month, respectively. Elective surgeries restarted following COVID-19 restrictions in July 2021 and corresponded with a spike in MyStay Cardiac usage (Figure 1). Figures 2 and 3 demonstrated that the most frequently accessed resources were information about the patients’ ICU stay, followed by information about their recovery on the ward and a recommended exercise program.

## Discussion

### Background

This study found that the use of digital multimedia resources to support patient education was well received and integrated into their practice by cardiac nurse educators working in acute care during the COVID-19 pandemic. Usage patterns fluctuated, reflecting variations in the number of elective cardiac surgery cases being treated at the study site over time. Data suggested a pattern of greater usage of the MyStay Cardiac during the COVID-19 pandemic when access to the health service for nonfrontline, essential workers was limited. Following the lifting of these restrictions, use of the MyStay Cardiac resources has been steady, reflecting ongoing use by the cardiac team for pre- and postoperative education. We acknowledge the possibility that the widespread diffusion of communications technologies during the COVID-19 pandemic may have increased patients’ capacity to interact with health technologies in the post–COVID-19 era [24]. Despite this, observed trends for decreased use of the MyStay Cardiac in the final year of the follow-up period may reflect that clinicians were able to provide more in-person education to patients following the lifting of COVID-19 restrictions or that the novelty of using a new digital multimedia resource had decreased with time. This finding highlights the need for ongoing updates of educational materials and new approaches to engaging staff to use these resources in their practice to sustain staff interest over the longer term.

The finding that approximately a third of access sessions involved users reviewing the website content more extensively and a quarter of sessions involved the in-depth use of the website suggested that use of the multimedia resources was acceptable to consumers. This finding is in line with recent research using digital multimedia and animation with adults undergoing chemotherapy treatment [25]. In this study, the most popular resources were the animation providing advice about “COVID-19 and Oncology care,” a general orientation to cancer care, and a video about receiving chemotherapy treatment.

The data on resource usage patterns and the length of time spent reviewing different sections of the program indicated that the cardiac nurses using the resource for patient education focused on information provision in relation to the patients’ ICU and ward stay, rather than focusing on engaging patients to participate in their care. Review of patient recovery goals and recommended exercises only accounted for approximately a third of resource usage, while accessing information about pain management and the patient pain record accounted for 6%-10% of usage.

The lack of focus on promoting patient participation in their recovery goals may highlight some of the disadvantages of using a passive diffusion approach to implementation [22]. Cardiac nurses integrated the MyStay resources into their own workflow efficiently but did not use the digital materials to promote patient engagement in line with the initial design of the app. Rather, their focus was on the effective communication of information rather than the promotion of patient participation [26]. These findings highlight that to achieve all the potential benefits of using the MyStay Cardiac resources in clinical care, further implementation needs to be supported by a structured approach to upskilling nurses working in acute care through the use of coaching and rapport-building communication techniques that promote greater patient participation in their care [27]. Developing nurses’ communication skills in partnering with consumers is particularly relevant with the increasing focus on developing comprehensive care plans that focus on achieving patient goals rather than clinician goals of care during an acute care admission [5].

The COVID-19 pandemic has accelerated the adoption of digital technology in health care settings [28]. A systematic review of literature published in 2020 (reflecting the height of pandemic-related health care activity worldwide) found 124 studies reporting the adoption of digital technology. Most studies reported digital technology use for diagnosis (n=64, 52.4%), surveillance (n=46, 37.1%), or prevention (n=37, 30.6%). Nine percent (n=11) of the included studies used digital technology to promote patient engagement in care [29]. During the pandemic, health service providers developed web-based platforms to facilitate patient engagement in a range of administrative tasks and access telehealth appointments [30]. However, the authors found that it was common for the level of sophistication of these websites and apps to insufficiently support patients in completing administrative tasks independently.

The use of electronic communication and digital communication technologies during COVID-19 was rapidly accepted by consumers from a diverse range of backgrounds, providing evidence that digital technology use in health care has been accelerated by the COVID-19 pandemic [31-33]. Cadel et al [31] conducted a scoping review of patient engagement activities used during the COVID-19 pandemic and found most activities focused on clinical interactions such as telehealth consultations, family visits, and community outreach using digital technology, and that most patients (>90%) were highly satisfied with their experiences of telehealth. Zeng et al [32] surveyed health care consumers in the United States on the use of electronic communication for health care before and during the COVID-19 pandemic and reported that the odds of technology use substantially increased during the COVID-19 pandemic (adjusted odds ratio 1.99, 95% CI 1.18-3.35). Individuals in the highest-income group were more likely to use technology than those in the lowest-income group. Despite this, individuals with lower educational attainment had similar growth in the use of electronic communication during the pandemic to those with postgraduate education.

### Strengths and Limitations

The strength of this study is that it provides long-term follow-up data demonstrating the acceptability and durability of implementing the use of digital multimedia resources to support patient education and participation in the acute care context. Considering its unusual “diffusion” approach to implementation, this research has provided baseline data of particular relevance to future investigations in the uptake of multimedia education resources and to assess future enhancements such as multilingual functions to reduce health inequalities in the accessibility of such resources. Study limitations are that only group level data on usage patterns was available for analysis from the study site, meaning that it was not possible to evaluate individual patient factors that may influence engagement and uptake of the My Stay Cardiac resources. Further research will focus on evaluating patient factors that influence engagement and further development and evaluation of the MyStay Cardiac using a co-design approach involving both the multidisciplinary clinical team and consumers. Future research would also benefit from randomized controlled studies to demonstrate the efficacy and effectiveness of the platform, and such studies are planned.

### Conclusions

The use of digital multimedia resources to support the education of patients undergoing cardiac surgery appeared to be well received by cardiac nurses and successfully integrated into usual practice during the COVID-19 pandemic. The acceptability of the MyStay Cardiac multimedia program to acute care nurses was demonstrated by sustained usage to support the provision of patient education over a 30-month follow-up period.

## Appendix

Multimedia Appendix 1 List of variables.

## Abbreviations

ICU: intensive care unit
