# Supplementary material for: Embedding the Use of Patient Multimedia Educational Resources Into Cardiac Acute Care: Prospective Observational Study
Source: JMIR Nurs. 2024 Jul 18;7:e54317. doi: 10.2196/54317 (PMC11294779; doi:10.2196/54317)
Supplement: Multimedia Appendix 1 [file nursing_v7i1e54317_app1.docx]

## Multimedia Appendix 1. List of Variables

| **Variable Name** | **Variable values** | **Description** |
| --- | --- | --- |
| Cohort | Nominal variable   - Year 1 (Aug 2020 – Jul 2021) - Year 2 (Aug 2021 – Jul 2022) - Year 3  (Aug 2022 – Jan 2023) | Grouping variable to run longitudinal comparisons  **note COVID restrictions in Year 1 & 2* – *reduction in elective surgeries* |
| Total number of usage sessions | Continuous variable (frequency) | Number of usage sessions. A ‘session’ is defined as a continuous block of use with inactivity < 15 mins. |
| Mean activity duration | Continuous variable (mins, secs) | Average duration viewed content before next click. |
| Total session duration | Continuous variable (mins, secs) | Total duration of usage session. |
| Session type | - Rapid views - Content exploration - In-depth look | **Rapid view** (session ≤ 2 minutes total duration, involving rapid sequence of clicks);  **Content exploration** (session > 2 minutes total duration, videos/audios previewed, but not completed); and  **In-depth looks** (session > 2 minutes total duration, videos/audios viewed to completion.  Values derived from content analysis of application usage data between August 2020 and September 2021 (see McDonall et al, 2022). |
| Day | Day of the week (Nominal variable) | Day of the week. Note that patient education often occurred on Wednesdays.  **note: surgeries occur of Thursday*  If Wednesdays frequent, analysed as a dummy variable -> Wednesday 0, 1 |
| Which_Domains accessed | Binary variables:   - ICU stay - Ward stay - Goals - Exercise - Healthcare team - Managing pain / pain records - Safety - Leaving hospital - Outpatient rehabilitation | Which parts of the website were accessed during each session? |
| Frq_Domains accessed | Continuous variables (frequency)   - ICU stay - Ward stay - Goals - Exercise - Healthcare team - Managing pain / pain records - Safety - Leaving hospital - Outpatient rehabilitation | How many times were different parts of the website accessed during each session? |
| Which Content accessed | Binary variables:   - Audio - Video - Text - Pain record | What types of media were accessed during each session? |
| Frq_Content accessed | Continuous variables (frequency):   - Audio - Video - Text - Pain record | How many times were different types of media accessed during each session? |
